# Ultramafic geoecology of South and Southeast Asia

**DOI:** 10.1186/s40529-017-0167-9

**Published:** 2017-04-03

**Authors:** M. L. Galey, A. van der Ent, M. C. M. Iqbal, N. Rajakaruna

**Affiliations:** 1 Center for Water and Environment, Natural Resources Research Institute, University of Minnesota, Duluth, MN 55811 USA; 2 Centre for Mined Land Rehabilitation, Sustainable Minerals Institute, The University of Queensland, Brisbane, QLD Australia; 3 Laboratoire Sols et Environnement, Université de Lorraine-INRA, UMR 1120, Nancy, France; 4 Plant Biology Laboratory, National Institute of Fundamental Studies, Kandy, 20000 Sri Lanka; 5 Biological Sciences Department, California Polytechnic State University, San Luis Obispo, CA 93407 USA; 6 Unit for Environmental Sciences and Management, North-West University, Potchefstroom, 2520 South Africa

**Keywords:** Adaptations, Conservation, Edaphic endemism, Edaphic flora, Extreme environments, Geobotany, Plant–soil relations, Serpentine vegetation, Ultramafic plants, Metal hyperaccumulators

## Abstract

Globally, ultramafic outcrops are renowned for hosting floras with high levels of endemism, including plants with specialised adaptations such as nickel or manganese hyperaccumulation. Soils derived from ultramafic regoliths are generally nutrient-deficient, have major cation imbalances, and have concomitant high concentrations of potentially phytotoxic trace elements, especially nickel. The South and Southeast Asian region has the largest surface occurrences of ultramafic regoliths in the world, but the geoecology of these outcrops is still poorly studied despite severe conservation threats. Due to the paucity of systematic plant collections in many areas and the lack of georeferenced herbarium records and databased information, it is not possible to determine the distribution of species, levels of endemism, and the species most threatened. However, site-specific studies provide insights to the ultramafic geoecology of several locations in South and Southeast Asia. The geoecology of tropical ultramafic regions differs substantially from those in temperate regions in that the vegetation at lower elevations is generally tall forest with relatively low levels of endemism. On ultramafic mountaintops, where the combined forces of edaphic and climatic factors intersect, obligate ultramafic species and hyperendemics often occur. Forest clearing, agricultural development, mining, and climate change-related stressors have contributed to rapid and unprecedented loss of ultramafic-associated habitats in the region. The geoecology of the large ultramafic outcrops of Indonesia’s Sulawesi, Obi and Halmahera, and many other smaller outcrops in South and Southeast Asia, remains largely unexplored, and should be prioritised for study and conservation.

## Background

Ultramafic soils are weathered products of lithologies, such as peridotite and serpentinite bedrock, consisting predominantly of ferromagnesian silicate minerals (Cardace et al. 2014; Moores 2011). Ultramafic soils are generally deficient in essential plant mineral nutrients (phosphorus, potassium), have major cation imbalances (low calcium-to-magnesium molar ratios), and have high concentrations of certain phytotoxic elements, including nickel, cobalt and manganese (Brady et al. 2005; Kazakou et al. 2008; O’Dell and Rajakaruna 2011). Tropical ultramafic soils, unlike those in temperate regions (Alexander 2009; Alexander and DuShey 2011), can be strongly weathered due to rainfall intensity and high temperature, and depending on elevation, can develop as laterites (e.g. Ferralsols) (Kruckeberg 2002; Mandal et al. 2015; van der Ent et al. 2013a; Vithanage et al. 2014).

Depauperate ultramafic soils may generate selective pressures promoting speciation and the evolution of ultramafic endemism (Anacker 2014; Kay et al. 2011; Rajakaruna 2004), often leading to distinctive plant communities worldwide (Anacker 2011; Brooks 1987). The biota of ultramafic soils has contributed greatly to the development of ecological and evolutionary theory (Harrison and Rajakaruna 2011; Strauss and Cacho 2013) and to the study of the genetics of adaptation and speciation (Brady et al. 2005; Palm and Van Volkenburgh 2014; von Wettberg and Wright 2011). Ultramafic floras are, however, threatened by deforestation, agricultural development, mining, and climate change-associated stressors (Boyd et al. 2009; Harrison et al. 2009; Rajakaruna and Boyd 2008; Vallano et al. 2012). These threats to ultramafic biota provide opportunities for conservation and restoration-oriented research (Elam et al. 1998; O’Dell and Claassen 2011; Weiss 1999; Whiting et al. 2004; Wolf 2001).

South and Southeast Asia contain several globally significant biodiversity hotspots (Mittermeier et al. 2005), including areas in Indo-Burma, Philippines, Sundaland (western half of the Indo-Malayan archipelago), and Western Ghats and Sri Lanka. The Borneo lowlands is the only ecoregion globally to surpass 10,000 plant species (Kier et al. 2005) and North Borneo is one of the top five biodiversity centres in the world (Barthlott et al. 2007). Despite South and Southeast Asia harboring several important biodiversity hotspots, the influence of edaphic factors on biodiversity is largely unknown (van der Ent et al. 2015a). Compared to research on ultramafic outcrops in temperate and Mediterranean regions (Alexander et al. 2007; Rajakaruna et al. 2009), ultramafic geoecology in this part of the world is also substantially understudied (Proctor 1992, 2003). In terms of tropical regions, most research related to ultramafic floras to date has focussed on New Caledonia (Isnard et al. 2016; Jaffré et al. 2010, 2013; Pillon et al. 2010; Pillon 2012). Although ultramafic outcrops of New Caledonia are of a similar latitude and general climate to South and Southeast Asia, the evolutionary histories of its flora and fauna are distinct. New Caledonia is on the east of the Lydekker’s Line, which separates the eastern edge of Wallacea from the Australian Region (which lies on the Sahul Shelf), marking a distinct change in floristic affinities. In this review, we also exclude New Guinea (Indonesian West Papua and Papua New Guinea) for the same reason, but note that despite the concomitant occurrence of ultramafic outcrops and exceptionally high biodiversity, virtually nothing is known about the ultramafic geoecology of this island. Research on the floristics and ecology of the understudied ultramafics of South and Southeast Asia is critical to provide a comprehensive assessment of the ultramafic geoecology of tropical Asia.

**Fig. 1:**
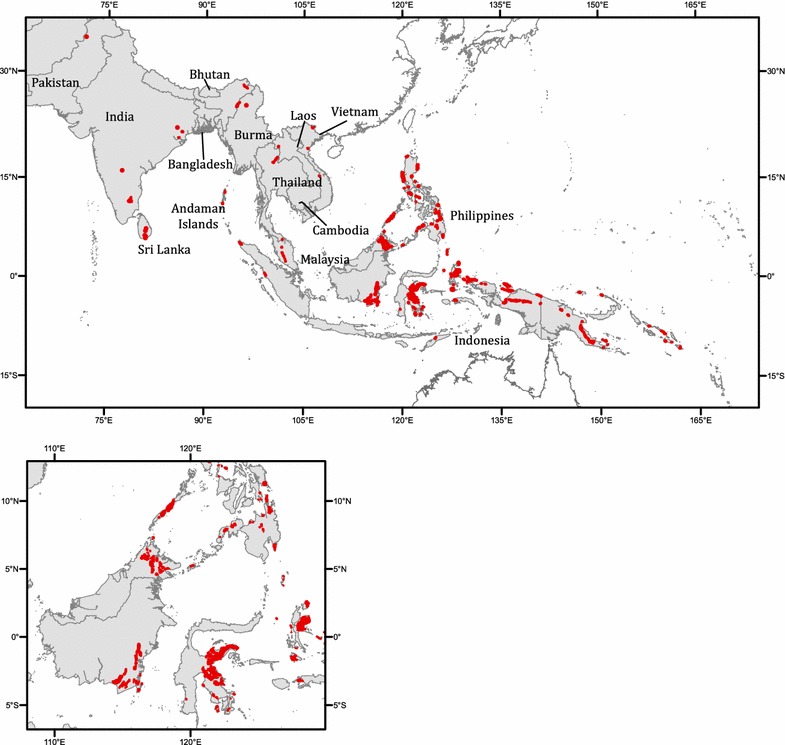
Map of South and Southeast Asia showing the distribution of ultramafic outcrops in the region. *Bottom inset* is a more detailed outline of ultramafic outcrops in Borneo, Palawan, Mindanao, Sulawesi, and Halmahera. Not all regions of India have complete geologic surveys, and we were unable to locate precise information about ultramafic outcrops in Burma and Laos. The ultramafic outcrop location in Northern Thailand is approximate. The extent of each outcrop shown is not to scale [Figure compiled with data from Central Energy Resources Team (1999), Datta et al. (2015), Kfayatullah et al. (2001), Shi et al. (2012), Baker et al. (1992), Van der Ent et al. (2013a, 2015a), Tan and Khoo (1993), MacDonald and Barr (1984), Geological Survey of India, Geological and Mineral Maps of States and Regions (http://www.portal.gsi.gov.in/portal/page?_pageid=127,603606&_dad=portal&_schema=PORTAL), and OneGeology Portal (http://portal.onegeology.org/OnegeologyGlobal/)]

This review examines the literature on the geoecology of ultramafic areas in South and Southeast Asia, covering India, Pakistan, and Sri Lanka to the west, Myanmar and Cambodia to the north, and Malaysia, Indonesia (excluding West Papua), and the Philippines to the east (Fig. 1; Table 1); all of which lie on the western side of Lydekker’s line and share a similar climate. We focus on (i) soil–plant relations, including studies on floristic diversity, soil–plant elemental relations, and soil microbes; (ii) ecological aspects, including studies on vegetation structure and composition and plant endemism; (iii) cross-kingdom interactions, including studies on herbivory, mycorrhizal associations, and invertebrate diversity; (iv) evolutionary aspects; (v) physiology and genetics; (vi) phytotechnologies; and finally, (vii) threats and conservation. We conclude the review by highlighting countries within South and Southeast Asia requiring further study, drawing attention to major gaps in knowledge.

**Table 1:** A summary of geoecological studies conducted on ultramafic outcrops in South and Southeast Asia

Country	Area of study	References
India	Bioremediation of chromite mines and nickel recovery by fungi	Acharya et al. (1998), Biswas et al. (2013), Bohidar et al. (2009), Ghosh and Paul (2015), Mishra et al. (2009)
	Discovery of nickel hyperaccumulators	Datta et al. (2015)
	Forest vegetation structure	Prasad et al. (2007)
	Heavy metal leaching into groundwater	Dhakate and Singh (2008)
	Heavy metal tolerance in ultramafic soil-associated microbes	Pal et al. (2004, 2005, 2006, 2007), Pal and Paul (2012)
	Origin and serpentinization of ultramafic rocks in the Indo-Myanmar subduction zone	Ningthoujam et al. (2012), Soibam et al. (2015)
	Phytoremediation of and bioaccumulation of metals from chromite mines	Mohanty et al. (2011, 2012)
	Plant–soil elemental relations on a chromite mine	Samantaray et al. (2001)
	Remote sensing for detecting and mapping ultramafic vegetation	Chaudhury et al. (2015)
	Ultramafic geology, geochemisty, mineral prospecting	Banerjee (1972), Chakraborty and Chakraborty (1984), Bhatta and Ghosh (2014), Mandal et al. (2015), Mitra (1973)
Indonesia	Acidification of serpentinite-derived soils	Fujii et al. (2011)
	Floristics and plant community structure	Proctor et al. (1994), van Balgooy and Tantra (1986)
	Geochemistry, petrography and thermobarometry of the ultramafics	Linthout and Helmers (1994)
	Nickel hyperaccumulators and phytotechnologies	Netty et al. (2012), van der Ent et al. (2013a)
	Species discovery on ultramafic soils	Cheek (2015)
Malaysia	Copper accumulation in ultramafic plants	van der Ent and Reeves (2015)
	Discovery of nickel hyperaccumulators	Hoffmann et al. (2003), van der Ent and Mulligan (2015), van der Ent et al. (2013b, 2016b, c)
	Ecology of nickel hyperaccumulators: nickel insects	van der Ent et al. (2015f)
	Floristics, plant–soil relations, ultramafic endemism	Chen et al. (2014), Fowlie (1985), Peng et al. (2015), Proctor et al. (1988a, b, 1989), Sugau and van der Ent (2016), van der Ent and Wood (2013), Wood and van der Ent (2012), Wong and van der Ent (2014), van der Ent and Wong (2015), van der Ent and Vanijajiva (2014)
	Metal localization; nuclear microprobe imaging analyses	Mesjasz-Przybylowicz et al. (2015)
	Ultramafic forest vegetation structure, plant ecology, community ecology	Adam (2002), Aiba et al. (2015), Aiba and Kitayama (1999), Brearley (2005), Bruijnzeel et al. (1993), Kitayama (1992), Proctor et al. (1988a, b), Sawada et al. (2015), Tashakor et al. (2013), van der Ent et al. (2015a, b, f, 2016a)
	Ultramafic geochemistry	Tashakor et al. (2011, 2013)
	Ultramafic plant–other biota interactions	Wells et al. (2011)
	Ultramafic-associated insects and soil invertebrates	Chung et al. (2013), Hasegawa et al. (2006), Jones et al. (2010), Leakey and Proctor (1987)
Myanmar	Mineralogy of jadeitite and related rocks, including serpentinites	Shi et al. (2012)
Pakistan	Ultramafic geochemistry and soil–plant metal relations	Kfayatullah et al. (2001), Naseem et al. (2009), Shah et al. (2010, 2014)
Philippines	Discovery of Ni hyperaccumulators	Baker et al. (1992), Fernando et al. (2014), Gotera et al. (2014), Hoffmann et al. (2003), Quimado et al. (2015)
	Herbivory on ultramafic soils	Proctor et al. (2000a)
	Metal tolerance in mycorrhizal fungi of ultramafic soils	Aggangan et al. (1998)
	Phytomining considerations	Fernando et al. (2013)
	Species discovery on ultramafic soils	Argent et al. (2007), Fernando and Rodda (2013), Fleischmann et al. (2011)
	Ultramafic forest vegetation structure and soil–plant relations	Bruijnzeel (1990), Proctor et al. (1997, 1998, 1999, 2000b)
	Ultramafic soil and forest litter invertebrates	Thomas and Proctor (1997)
Sri Lanka	Antimicrobial activities of ultramafic-associated plants	Rajakaruna et al. (2002)
	Ecotypic differentiation of ultramafic taxa	Chathuranga et al. (2015)
	Phyto- and bio-remediation of ultramafic soils; soil remediation	Bandara et al. (2017), Herath et al. (2014), Kumarathilaka et al. (2016), Seneviratne et al. (2016a, b)
	Soil–plant relations including floristics, soil–plant elemental relations, discovery of nickel and copper hyperaccumulators	Brooks (1987), Rajakaruna and Baker (2004), Rajakaruna and Bohm (2002), Samithri (2015), Senevirathne et al. (2000), Weerasinghe and Iqbal (2011)
	Ultramafic geology and geochemistry	Dissanayaka (1982), Dissanayake and Van Riel (1978), Munasinghe and Dissanayake (1980), Hewawasam et al. (2014), Rajapaksha et al. (2012, 2013), Ranasinghe (1987), Tennakoon et al. (2007), Vithanage et al. (2014)
Southeast Asia: Regional Overviews	Floristics, plant–soil elemental relations, metal accumulators, quantitative bedrock (including ultramafic) geology of Southeast Asia	Brooks (1987), Brooks et al. (1977a, b), Brooks and Wither (1977), Peucker-Ehrenbrink and Miller (2004), Proctor (1992, 2003), Reeves (2003), van der Ent et al. (2015c, d), Wither and Brooks (1977)
Thailand	Petrography and geochemistry of ultramafic rocks	Hisada et al. (2004), Macdonald and Barr (1984), Orberger et al. (1995)
Vietnam	Heavy metal (Cr, Ni, Co) leaching from chromite mine	Kien et al. (2010)
	Ultramafic geology	Thanh et al. (2014)

Information within columns organized in alphabetical order

## Soil–plant relations

Ultramafic soils worldwide share a distinct suite of chemical and physical features (Rajakaruna et al. 2009); however, tropical ultramafic soils may differ in elemental content, moisture, organic matter content, and soil pedology (Kierczak et al. 2007; Vithanage et al. 2014), compared to those in temperate and Mediterranean regions (Alexander 2009; Alexander et al. 2007). Table 2 lists key soil properties of ultramafic soils from South and Southeast Asia, focusing on pH, Ca:Mg molar ratio, Ni, Cr, and the major nutrients, P and K. Plants growing on ultramafic soils have to contend with a suite of edaphic stressors, including low nutrient content, high levels of phytotoxic elements, and, at times, water stress (Brady et al. 2005). Plants and soil microbes of ultramafic soils tolerate these edaphic stressors via efficient uptake of essential nutrients, and exclusion of, or conversely accumulation and localization of high concentrations, of certain phytotoxic elements, among other adaptations (see Palm and Van Volkenburgh 2014 for a discussion).

**Table 2:** Selected soil chemical properties of ultramafic outcrops in South and Southeast Asia

Country	Altitude (masl)	pH	Ca:Mg	Ca (exch.) cmol (+) kg^−1^	Mg (exch.) cmol (+) kg^−1^	K (exch.) cmol (+) kg^−1^	K (μg g^−1^)	P (μg g^−1^)	P (extract.) μg g^−1^	Ni (μg g^−1^)	Ni (extract.) μg g^−1^	References
Sulawesi, Indonesia	–	5.3–6.3	0.9–5.7	4.6–13.3	11.1–26.2	0.05–0.5	–	–	–	825–4050	–	Parry (1985)
Sulawesi, Indonesia	200–300	5.8–7.0	0.1–1.6	0.2–1.6	0.5–4.6	0.01–0.1	3281–6260	14.4–237	0.23–3.87	3730–10,524	2.1–30.2	Van der Ent et al. (2013a)
Talaud Island, Indonesia	60–500	6.1–6.4	1.6–32	0.9–16	13.9–27.3	0.19–0.38	–	–	0.94–6.8	–	8.5–37	Proctor et al. (1994)
Sibuyan Island, Philippines	325–1540	4.3–5.5	0.3–2.9	0.5–3.4	0.75–3.64	0.04–0.41	–	–	0.41–2.07	–	1–24	Proctor et al. (1998)
Palawan, Philippines	50	6.8	0.24	4.27	18.1	0.32	–	–	1.02	6900	360	Proctor et al. (1999)
Sabah, Malaysia	400–2900	3.8–9.7	0.1–136	0.003–35	0.02–76	0.002–0.79	0.1–1056	4.4–585	0.1–32	17–9308	0.17–442	Van der Ent (unpublished)
Sabah, Malaysia	180	5.3	0.62	0.86	1.38	0.17	–	201	0.34	2980	10.8	Brearley (2005)
Sabah, Malaysia	280	5.7	0.31	7.7	24.6	0.14	–		4.1	–	–	Proctor et al. (1988a)
Ussangoda, Sri Lanka	15–20	5.3–6.2 (4.3–4.9)	0.6–1.9 (1.4–2.4)	187–905^a^ (112–212)	311–456^a^ (60–122)	–	140–321 (163–350)	–	–	–	101–151 (29–65)	Weerasinghe and Iqbal (2011), Rajakaruna and Bohm (2002)
Ginigalpalessa, Sri Lanka	70–80	5.7–7.4	0.1–0.6	180–1580^a^	2400–3400^a^	–	70–230	–	–	–	15–180	Rajakaruna and Bohm (2002)
Indikolapalessa, Sri Lanka	70–80	4.7–6.1	0.2–2.6	395–1863^a^	613–2625^a^	–	78–1563	–	–	–	4–148	Rajakaruna and Bohm (2002)
Yodhagannawa, Sri Lanka	90–100	5.1–5.7	0.1–0.2	123–138^a^	838–1000^a^	–	53–75	–	–	–	47–79	Rajakaruna and Bohm (2002)
Andaman, India	50–732	6.0–6.8	–	–	2300–3600^a^	–	–	–	–	3370–9030	397–913	Pal et al. (2007)
Andaman, India	50–732	4.4–7.1	–	–	2.8–3.9^b^	–	–	–	–	244–10,107	192–907	Datta et al. (2015)

Units are listed under each soil variable except for values with superscripts: ^a^ μg g^−1^; ^b^ %

### Plant diversity and soil–plant elemental profiles

In Sukinda, India, chromite mine spoils composed of ultramafic substrates have Ni ranging from 187 to 215 µg g^−1^ and Ca:Mg molar ratios of 1.69–2.27; from which, in total, 113 plant species belonging to 51 families have been recorded (Samantaray et al. 2001). Some species which colonize the substrate exhibit traits typical of plants adapted to ultramafic soils, including sclerophyllous and microphyllous leaves (Brady et al. 2005), but individual plants also show chlorosis, leaf curling, and necrosis.

On the Andaman Islands, India, ultramafic soils with high Ni concentrations (2700–10,100 μg g^−1^) harbor eight Ni hyperaccumulator plant species belonging to eight different genera and seven different families (Datta et al. 2015). Of these, *Dichapetalum gelonioides* subsp. *andamanicum* (Dichapetalaceae) and *Rinorea bengalensis* (Violaceae) accumulated up to 30,000 μg g^−1^ Ni. There is substantial potential for using remote sensing tools to examine the vegetation communities on the ultramafics of the Andaman Islands, where the ultramafic outcrops are mostly inaccessible and the vegetation deserves more intensive exploration (Chaudhury et al. 2015).

In Northern Pakistan, the ultramafics of Mingora and Kabal in the Swat region include assemblages of serpentinite, green schist, talc-carbonate schist, and metabasalts in the Mingora–Shangla mélange zone (Shah et al. 2010). Relatively high accumulation of Ni and Cr has been recorded in the plant tissue of *Indigofera gerardiana* (Fabaceae), *Saccharum griffithii* (Poaceae), *Lycopersicon esculentum* (Solanaceae), and *Chrysopogon zizanioides* (Poaceae) growing in the Kot Parang Ghar mélange zone in the Bucha Area, Pakistan (Shah et al. 2010, 2014).

In Sri Lanka, ultramafic rocks occur along a Precambrian suture zone at the boundary of the Vijayan and Highland Series, metamorphic remnants of two ancient tectonic plates (Dissanayaka 1982; Munasinghe and Dissanayake 1980). The geochemistry of these outcrops, particularly of Ussangoda along the southern coast, has been well-documented (Hewawasam et al. 2014; Rajapaksha et al. 2012, 2013; Tennakoon et al. 2007; Vithanage et al. 2014). The floristics of the ultramafic outcrops of Sri Lanka, especially of Ussangoda, have also received considerable attention (Brooks 1987; Rajakaruna and Baker 2004; Rajakaruna and Bohm 2002; Rajakaruna et al. 2002; Samithri 2015; Senevirathne et al. 2000; Weerasinghe and Iqbal 2011).

Research suggests that Sri Lanka’s ultramafic flora is impoverished with respect to the total number of plant species and percent proportion of endemic species. To date, 67 plant species belonging to 61 genera and 30 families have been identified from Ussangoda (Samithri 2015). Combined with an additional 40 taxa reported from three other sites surveyed by Rajakaruna and Bohm (2002), the total ultramafic flora of Sri Lanka stands at a mere 107 species, compared to many-fold more documented from other sites in Southeast Asia (van der Ent et al. 2015a). Of the species documented from ultramafic soils, only *Vernonia zeylanica* (Asteraceae) is endemic to Sri Lanka (MOE 2012), although the taxon is not restricted to the substrate.

### Soil microbes

Several recent studies, conducted in temperate and Mediterranean regions of the world, explore the roles microbes play in the ecology of ultramafic habitats as well as in the remediation of metal-contaminated soils (Batten et al. 2006; Ma et al. 2015; Schechter and Branco 2014). Although studies on microbial ecology of ultramafic soils in South and Southeast Asia are minimal, Pal et al. (2004, 2005, 2006, 2007) and Pal and Paul (2012) have carried out a series of studies on microbial diversity and ecology of ultramafic soils on the Andaman Islands, India. In one of these studies, Pal et al. (2005) compared physicochemical and microbial properties of ultramafic soils with those from adjacent non-ultramafic localities. The elemental profiles were characteristic of ultramafic soils, with high concentrations of Mg, Ni, Cr, and Co. Furthermore, the ultramafic soils showed low microbial density (6.2–11.3 × 10^6^ colony forming unit/g soil) and activity (1.7–3.5 µg fluorescein/g dry soil/h) relative to non-ultramafic soils. The ultramafic-associated microbial population (including bacteria and fungi) was dominated by bacteria and was more resistant to metals than populations from non-ultramafic soils. Among the ultramafic isolates, 8 and 11 bacteria tolerated >12.0 mM Ni and >16.0 mM Cr, respectively, while six fungal isolates showed a minimum inhibitory concentration (MIC) value >8.0 mM Co. The ultramafic strains also showed co-resistance to Cu, Zn, and Mn. Pal et al. (2007) also examined the soil microflora associated with the rhizosphere of two known Ni hyperaccumulators from the Andaman Islands, *R. bengalensis* and *D. gelonioides* subsp. *andamanicum*. Of the total 123 microbes (99 bacteria and 24 fungi) that were isolated, bacteria were more tolerant of Ni than fungi, showing their greater potential for Ni tolerance.

In a study focusing on medicinal qualities of wild-harvested plants, 32 plant species collected from ultramafic outcrops of Sri Lanka were screened for antimicrobial properties (Rajakaruna et al. 2002). Of these, 29 species belonging to 12 families proved effective against at least one microorganism. Photoactivity was also observed from extracts of 10 species belonging to 10 families. There was no observed correlation between trace element hyperaccumulation (Rajakaruna and Bohm 2002) and antimicrobial activity.

## Ecological aspects

Ultramafic outcrops have long-provided model settings for studies on the ecology of plant species and plant communities. Studies range from those investigating aspects of the ecology of edaphically specialized plant populations and plant–plant interactions to those exploring factors and mechanisms driving the assembly of plant communities (see Harrison and Rajakaruna 2011). Compared to other regions of the world, ecological studies on ultramafics of South and Southeast Asia are mostly limited to those examining floristics, plant community structure, and edaphic-floristic associations.

### Vegetation structure and composition

Mount Silam in Sabah, Malaysia, has been extensively studied, including the general floristics, forest structure, hydrology and chemical analysis of tree foliage and leaf litter (Proctor et al. 1988a, b, 1989; Bruijnzeel et al. 1993). The study plots on Mount Silam range from 280 to 870 masl in elevation, documenting a broad spectrum of vegetation changes with altitude. The site is extremely species-rich in terms of its tree flora, ranging between 19 species in a 0.04-ha plot at 870 masl to 104 species in a 0.4-ha plot at 480 masl (Proctor 1992). Ultramafic-associated rainforests on Mount Guiting-Guiting, Sibuyan Island, Philippines (Proctor et al. 1998) and those of Mount Silam, Sabah (Proctor et al. 1988a, b) are similar in their soil features (Ni, Ca:Mg, and depth) and lack of stunted lowland forests. At these locations, small-statured forests are associated with higher elevations.

On Mount Bloomfield in the western Philippines (Palawan), Proctor et al. (1999) described a very different forest structure from those of Mount Silam and Mount Guiting-Guiting. The soil depths on Mount Bloomfield are much less compared to these other sites; Bruijnzeel (1990) suggested that drought in the shallow soils is a major cause of forest stunting on ultramafics, perhaps in association with fire (Proctor et al. 1997). Mount Bloomfield lacks tall forests and instead is characterised by trees less than 18 m tall. No statistical relationship could be established between tree height and soil chemistry, although Proctor et al. (1999) did find a direct proportional relationship between maximum tree height and soil water retention. The authors indirectly linked soil water to fire susceptibility in establishing the particular vegetation pattern on Mount Bloomfield, one that superficially resembles fire-dependent vegetation of New Caledonia.

Proctor et al. (2000a, b) compared vegetation on ultramafic soils to those on non-ultramafic (greywacke-derived) soils in Palawan and found that the species richness and diversity of ultramafic and greywacke sites were similar. However, the individual species and familial composition were rather different, with only members of the Saxifragaceae occurring on both ultramafic and greywacke soils. Trees on the serpentinized peridotite had a high proportion of microphyllous leaves, which is not a general feature of ultramafic forests in the region. Differences in water supply and fire frequencies, in combination with edaphic difference, may contribute to the distinct forests overlying these soils (Proctor et al. 1999, 2000a, b).

Sulawesi and Halmahera in Indonesia have 15,400 and 8000 km^2^ of ultramafic outcrops, respectively (van der Ent et al. 2013a). Lateritic soils overlaying the bedrock harbor both sclerophyllous ultramafic vegetation and more cryptic tropical rainforest, which are nonetheless inhabited by a high proportion of endemic flora. Proctor et al. (1994) examined the ultramafic soil–plant relations of Mount Piapi on Karakelong part of the Talaud Islands, North Sulawesi, Indonesia and reported that the short stature of the local vegetation is a result of low water-holding capacity of the soil, while the unusual species assemblage likely results from the soil chemistry typical of ultramafic soils. They also documented an undescribed Ni-hyperaccumulating species of *Rinorea* from their study site.

Kinabalu Park, Sabah, one of the world’s most species-rich hotspots with more than 5000 plant species recorded in an area of just 1200 km^2^, is also home to extensive ultramafic exposures (van der Ent et al. 2014). Plant diversity on ultramafics of the Park decreases with elevation, with a mid-elevation (circum 1500 masl) ‘hump’ occurring for some plant groups (Orchidaceae, Pteridophytes) resulting from the presence of cloud forests (van der Ent et al. 2016a). Six main vegetation classes with associated soil types are described by van der Ent et al. (2016a), including Sub-Alpine Scrub and Graminoid Scrub, both associated with Hypermagnesic Cambisols (‘hypermagnesian soils’), Montane Cloud Forest, associated with Cambisols often with accumulation of humus, Mixed Dipterocarp Forest, associated with deep Ferralsols (‘laterites’), and Pioneer Casuarina Scrub and Mature Mixed Casuarina Forest, both associated with Hypermagnesic Leptosols. The ‘adverse’ soil chemistry exacerbates vegetation stunting but no clear correlation between elevation, soil chemistry and plant diversity was found, as some of the most ‘adverse’ soils on the summit of the entirely ultramafic Mount Tambuyukon (2359–2534 masl) had up to 132 species per 250 m^2^ (van der Ent et al. 2016a).

**Fig. 2:**
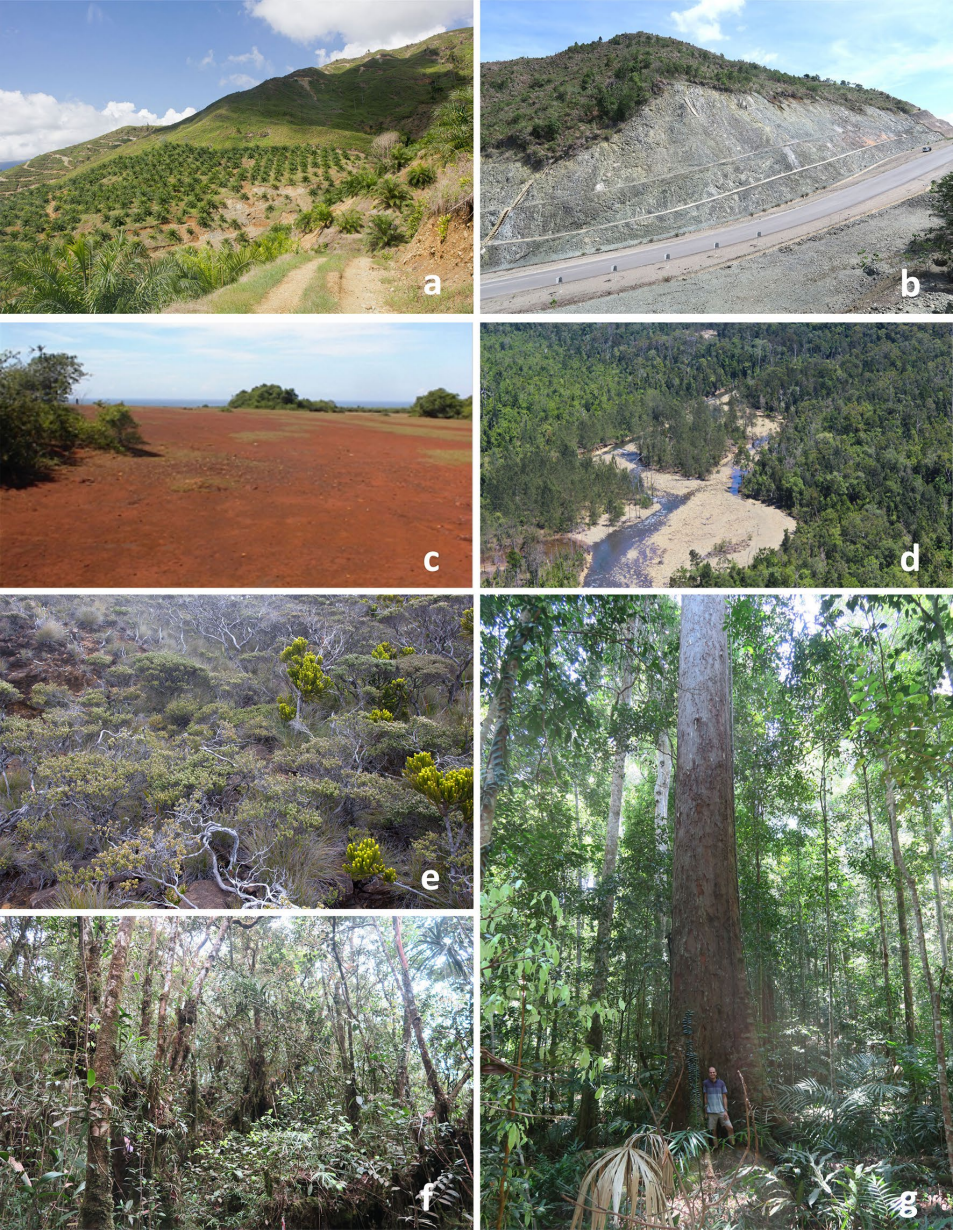
Ultramafic outcrops and vegetation in South and Southeast Asia: **a** Oil palm estate in Sabah, Malaysia on eroding ultramafic soils. **b** Road cut through strongly serpentinised bedrock in Sabah, Malaysia. **c** Bare red Ferralsols at Ussangoda in Sri Lanka. **d** River flowing through an ultramafic outcrop in Halmahera, Indonesia. **e** Extremely stunted sub-alpine vegetation on ultramafic soils in Kinabalu park, Malaysia. **f** Montane cloud forest on ultramafic soils on Mount Silam, Malaysia. **g** Exceptionally tall lowland mixed dipterocarp forest on ultramafic soils in Sabah, Malaysia (all images are by A. van der Ent, except **c** by Y.A.S. Samithri and **g** by Isabella Zelano)

Samithri (2015) examined the vegetation community composition and patterns at Ussangoda, Sri Lanka’s most intensively studied ultramafic outcrop. She found a higher diversity of plant species in ‘forest islands’ compared to the extensive ‘plains’ characterizing the site (Fig. 2c). Although the plains make up over 90% of the outcrop area, they only harbor 18 herbaceous species belonging to 17 genera and 11 families compared to 49 tree, shrub, herb and climber species belonging to 44 genera and 27 families found in the ‘forest islands.’ Although the soil chemical features did not differ significantly between sites on the ‘plains’ versus those in the ‘forest islands,’ soil features such as depth and resulting water holding capacity in ‘forest islands’ may favor the growth of a wide range of species than on the exposed and shallow soils of the ‘plains.’

Studies on bryophytes, lichens, and epiphytes on ultramafic outcrops are sparse worldwide (but see Boyd et al. 2009; Briscoe et al. 2009; Favero-Longo et al. 2004; Rajakaruna et al. 2012). In South and Southeast Asia, such studies are mostly non-existent. However, one study from the Philippines (Proctor et al. 2000b) documents epiphytic plants on trees of ultramafic and adjacent greywacke soils. The trees on the greywacke had fewer lianas and much less bole bryophyte cover than those on the serpentinized peridotite. Forty-one percent of trees on peridotite had >10% bryophyte cover, while none of the trees on greywacke soils had >10% bryophyte cover. The greywacke soils also hosted significantly higher densities of ferns, Cyperaceae spp., rattans (Arecaceae: Calamoideae), and Pandanaceae spp. compared to ultramafic soils, while ultramafic soils harbored significantly more herbaceous and bamboo (Poaceae: Bambusoideae) species. Floristic differences between the sites were attributed to differences in geochemistry, hydrology, and fire-frequencies (Proctor et al. 1999, 2000b).

### Plant endemism

Ultramafic soils, often with disproportionately high numbers of endemic species (Anacker 2011), are prime settings to explore the nature of edaphic endemism (Rajakaruna 2004). In New Caledonia, 2150 species occur on ultramafic soils of which 83% are restricted to these soils (Jaffré 1992; Jaffré and L’Huillier 2010), whereas in Cuba, 920 species (approximately one-third of the taxa endemic to Cuba) are found exclusively on ultramafic soils (Borhidi 1992). Similar restrictions and notable floristic associations are also found on ultramafic outcrops of Mediterranean climates (including California; Alexander et al. 2007; Safford et al. 2005), as well as in South Africa/Zimbabwe and Australia (Anacker 2011; Brooks 1987).

The restriction of habitat specialists to ultramafic soils is generally considered a consequence of their inherent slow growth rates that leads them to being outcompeted on more favorable soils (Anacker 2014; Anacker et al. 2011; Kay et al. 2011). Although some growth experiments have shown that habitat specialists can grow faster on more nutrient-rich soils (Kruckeberg 1954), species from the ultramafic maquis in New Caledonia have inherently slow growth, albeit becoming larger under more fertile conditions (Jaffré 1980). Table 3 lists the countries within the South and Southeast Asian region with ultramafic floras, including the number of ultramafic-associated species documented and the number of ultramafic endemics described in each country.

**Table 3:** Surface area covered by ultramafic rocks, total number of species in the regional flora, number of ultramafic-associated species, and number of ultramafic endemic species along with percent ultramafic endemism in the region’s flora for a number of global hotspots for ultramafic endemism and for regions within South and Southeast Asia

Region	Surface area of ultramafics (km^2^)	Total number of vascular plant species in the flora	Number of ultramafic-associated species	Number of ultramafic endemic species (% ultramafic endemism)	References
New Caledonia	5470	3371	2150	1785 (83)	Jaffré (1992), van der Ent et al. (2015d), Isnard et al. (2016)
California, United States	~6000	5271	492	246 (4.7)	Anacker et al. (2011), Burge et al. (2016), Jepson Flora Project (2016), Safford et al. (2005)
Queensland, Australia	818	8500	553	18 (0.2)	Batianoff and Specht (1992), Batianoff and Singh (2001)
Western Australia, Australia	5654	~12,000	1355	14 (0.12)	Van der Ent et al. (2015d)
New Zealand	~310	2418	~800	15 (0.6)	Lee (1992), New Zealand Plant Conservation Network (2016), Van der Ent et al. (2015d)
Cuba	5300	6375	–	920 (14)	Reeves et al. (1999)
Zimbabwe	~3000	6385	322	322 (5)	Wild (1965), Proctor and Cole (1992)
Sabah	~3500	~8000	4252	347 (4.3)	Van der Ent et al. (2015a)
Sulawesi, Indonesia	~15,400	~5000	na	na	Van der Ent et al. (2013a)
Palawan, Philippines	~3000	1522	na	na	Davis and Heywood (1995)
Sri Lanka	7	3492	107	0	MOE (2012), Rajakaruna and Bohm (2002), Samithri (2015)

In Sabah, Malaysia, *Borneodendron aenigmaticum* (Euphorbiaceae) is one of the few large rainforest trees restricted to ultramafic soils (Proctor et al. 1988a). Van der Ent and Wood (2012, 2013) describing orchid species associated with ultramafics in Sabah, Malaysia, documented many endemic species (Orchidaceae) restricted to narrow valleys with steep slopes, dominated by *Gymnostoma sumatranum* (Casuarinaceae) and *Ceuthostoma terminale* (Casuarinaceae). Further, van der Ent et al. (2015b) show habitat partitioning among ultramafic endemic *Nepenthes* species (Nepenthaceae) of Mount Kinabalu and Mount Tambuyukon, with distinct habitats and elevation ranges for the different *Nepenthes* taxa. *Eriobotrya balgooyi* (Rosaceae) was described as a new species restricted to ultramafic soils on a hill near the eastern ridge of Mount Kinabalu and on the nearby Mount Tambuyukon in Sabah, Malaysia (Wong and van der Ent 2014). The importance of scientific exploration of the ultramafics of Southeast Asia cannot be stressed enough; a survey on the ultramafic Mount Guiting-Guiting, Philippines (Argent et al. 2007) also led to the discovery of a new species, *Lobelia proctorii* (Campanulaceae).

Sri Lanka’s ultramafic outcrops and their flora, compared with ultramafic floras of Southeast Asia and Australia-Pacific region (van der Ent et al. 2015c, d), have received relatively little attention partly because they do not harbor any endemic species nor many metal hyperaccumulators (Chathuranga et al. 2015). All species so far documented from the ultramafic outcrops of Sri Lanka also have non-ultramafic populations, and it is unclear whether the ultramafic populations are physiologically distinct (i.e. ecotypes).

## Cross-kingdom interactions

Edaphically stressful substrates, like ultramafic soils, present plants with challenges that differ from more ‘benign’ substrates. Growing under such stress, ultramafic plants will likely encounter other organisms (herbivores, pathogens, beneficial insects and pathogens) that are also able to tolerate some of the same stressors affecting the plants (Strauss and Boyd 2011). There is evidence to suggest that pressures from enemies will be greater on edaphically stressful substrates than on normal soils (Strauss and Cacho 2013). Additionally, the enriched concentrations of certain trace elements, such as nickel, found in ultramafic soils may provide plants with opportunities for elemental defence (Boyd 2014). A significant body of research exists on plant–other biota interactions on ultramafic soils from temperate and Mediterranean climes, including studies on elemental defence (Boyd 2009), defence against pathogens (Hörger et al. 2013; Springer 2009), herbivory (Lau et al. 2008), mycorrhizal associations (Southworth et al. 2014), plant–pollinator interactions (Meindl et al. 2013; Wolf and Thorp 2011), and seed dispersal (Spasojevic et al. 2014). However, such studies are minimal in tropical Asia.

### Herbivory

In the only known published study on herbivory in ultramafic ecosystems in the region, Proctor et al. (2000a) found that the percentage of leaf area consumed was similar for plants found on and off of ultramafic soils on Mount Bloomfield, Palawan (Philippines), although the actual leaf area consumed was greater for the ultramafic forest as it had plants with larger leaves. There was no relationship between herbivory and leaf elemental chemistry; even the metal-accumulating taxa were attacked by herbivores. Proctor et al. (2000a) speculate that the gall-forming and leaf-mining insects must be tolerant of nickel as they spend their entire juvenile stage in the leaf tissue.

**Fig. 3:**
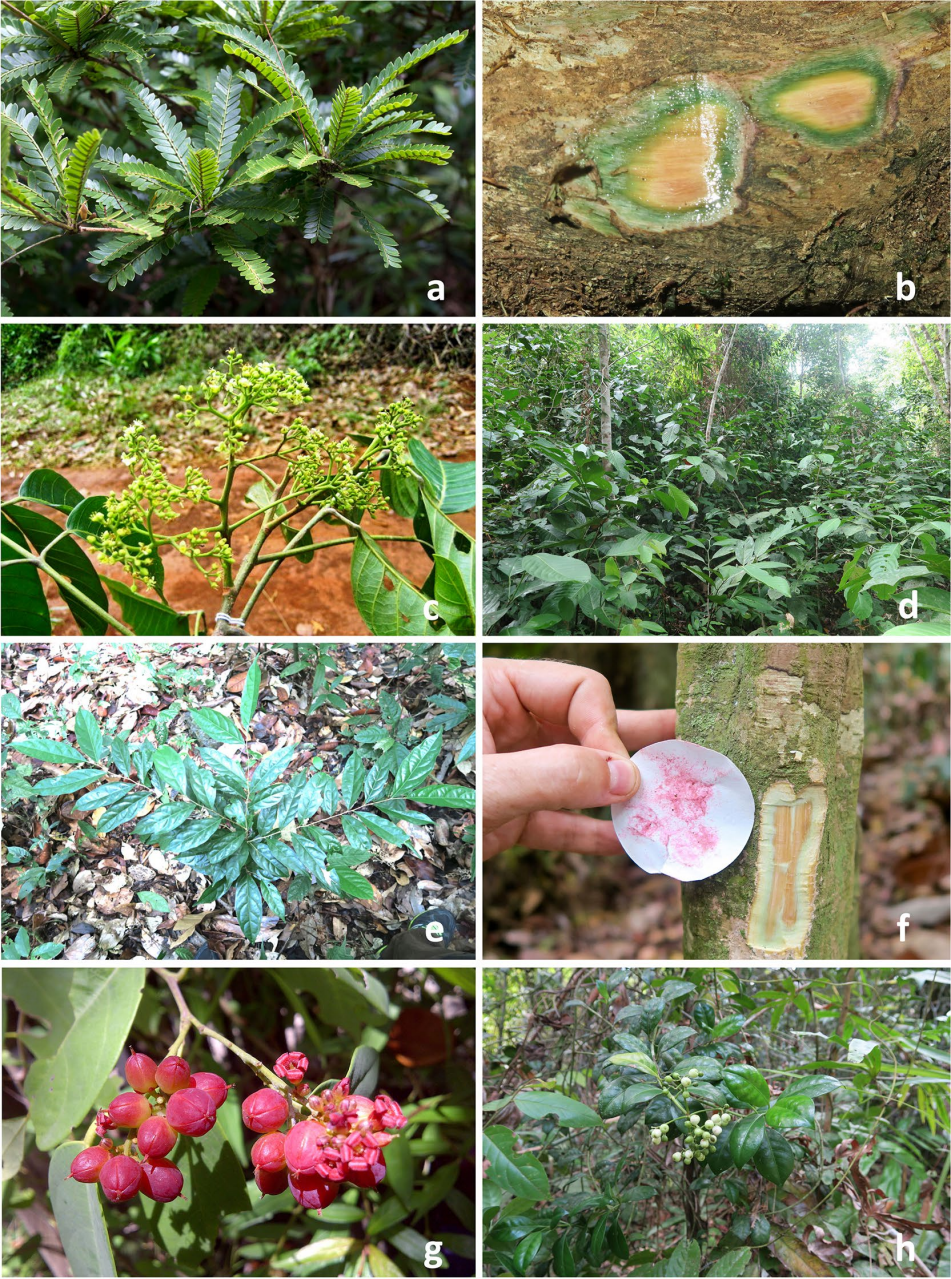
Nickel hyperaccumulator plants in South and Southeast Asia: **a** *Phyllanthus balgooyi* (Phyllanthaceae) in Sabah, Malaysia is a small understorey tree. **b** Phloem sap exuding from *Phyllanthus balgooyi* contains up to 20 wt% Ni. **c** *Knema matanensis* (Myristicaceae) in Sulawesi, Indonesia; **d** *Rinorea bengalensis* (Violaceae) can be locally dominant in lowland forest, in Sabah, Malaysia. **e** *Dichapetalum gelonioides* subsp. *tuberculatum* (Dichapetalaceae) from Mount Silam, Malaysia. **f** Main stem of *Dichapetalum gelonioides* subsp. *tuberculatum* showing its Ni-rich phloem tissue with colorimetric response in dimethylglyoxime test-paper. **g** *Sarcotheca celebica* (Oxalidaceae) from Sulawesi, Indonesia. **h** *Psychotria sarmentosa* (Rubiaceae) is the only known Ni hyperaccumulator in South and Southeast Asia that is a climber (all images are by A. van der Ent, except **c**, **g** are by A. Tjoa, Tadulako University, Indonesia)

Recent work by van der Ent and Mulligan (2015) show Ni accumulation in various parts of Ni hyperaccumulator plants occurring in Sabah, Malaysia, with the highest Ni concentration recorded in the phloem tissue (up to 7.9% in *R. bengalensis*) and phloem sap (up to 16.9% in *Phyllanthus balgooyi*); Ni localization in phloem tissue is visible by the bright green coloration in field-collected samples (Fig. 3b, f). The discovery of toxic levels of Ni in the phloem tissue suggests that the increased Ni in the phloem provides a defence against phloem-sap feeding insects, pathogens, and other herbivores (Boyd 2014; Hanson et al. 2004). However, Geometric moth larvae (Erebidae: Erebinae:Poaphilini) were found feeding on the leaves of the Ni hyperaccumulator *P. balgooyi*, furthermore aphids were found feeding on *Phyllanthus* cf. *securinegioides* (van der Ent et al. 2015f).

### Mycorrhizal associations

*Pisolithus tinctorius* (Sclerodermataceae), an ectomycorrhizal fungus, is found in the rhizosphere of *Eucalyptus urophylla* (Myrtaceae) from ultramafic soils in the Philippines, New Caledonia, and Western Australia (Aggangan et al. 1998). *Pisolithus tinctorius* was cultured with *E. urophylla* to determine the effects of Cr and Ni on the fungal growth rate. The fungus concentrates metals in the extramatrical hyphae and extra-hyphal slime and is particularly tolerant of high concentrations of Ni and Cr. There was geographic variation in terms of metal tolerance in the fungus, with the New Caledonian isolate outperforming both the Australian and the Philippines isolates. The Philippines isolate grew well on agar in the presence of Cr up to 2000 µmol L^−1^ and Ni up to 200 µmol L^−1^, but formed fewer mycorrhizae in vitro and in vivo than its counterparts from New Caledonia and Western Australia.

### Soil invertebrates

A study comparing termite assemblages on ultramafic-derived forest soils to those on non-ultramafic soils in Borneo, Malaysia shows that ultramafic sites have low species density (<35%), low relative abundance (<30%), a virtual absence of soil-feeders, significantly fewer wood-feeders, and a near-absence of species of Rhinotermitidae, *Amitermes*-group, *Termes*-group, *Pericapritermes*-group and *Oriensubulitermes*-group (Jones et al. 2010). The authors suggest that metal toxicity, high pH disrupting gut physiology, metal poisoning of essential microbiota in the termite gut, and metal bioaccumulation by gut microbes with subsequent poisoning of the termite host, as possible reasons for the depauperate termite communities on ultramafic soils.

A study on the patterns of Oribatid mite communities in relation to elevation and geology on the slopes of Mount Kinabalu, Sabah, Malaysia, shows that the density and morphospecies richness of Oribatid mites are greater in non-ultramafic soils than in the ultramafic soils at each of the same elevations (Hasegawa et al. 2006). The density and richness of Oribatid mites decreased with elevation on both substrates, but the effects of elevation on their density in non-ultramafic soil were less significant than in the ultramafic substrate.

An investigation of the invertebrate communities in forest litter and soil on Mount Guiting-Guiting in the Philippines, shows that ultramafic soils, even at higher elevations, were not poor in soil invertebrates, including Oligochaeta (Thomas and Proctor 1997), similar to earlier findings on Mount Silam, Sabah (Leakey and Proctor 1987).

## Physiology and genetics

There is considerable interest in understanding the physiology and the underlying genetic basis for traits conferring adaptation to ultramafic soils (Bratteler et al. 2006; Palm and Van Volkenburgh 2014; von Wettberg and Wright 2011; Wu et al. 2008). The advent of novel molecular methods has provided unique approaches to exploring stress tolerance (Selby et al. 2014; Visioli and Marmiroli 2013) and ultramafic-associated plants will continue to provide model systems for such investigations (Arnold et al. 2016; von Wettberg et al. 2014). While these advances have not yet been made in tropical Asia, the region provides numerous opportunities for investigating the physiological and genetic aspects of adaptation to ultramafic soils. To date, much of the research in South and Southeast Asia has focused on discovering new hyperaccumulating plant species from ultramafic soils in the region.

### Trace element hyperaccumulation

Plants found on ultramafic soils have long-been recognized as model systems to explore trace element hyperaccumulation (Gall and Rajakaruna 2013). There are well over 450 Ni hyperaccumulator plant species globally, all occurring on ultramafic soils (van der Ent et al. 2013c). Ultramafic associated plants are known to hyperaccumulate cobalt (Co) and Cu (>300 μg g^−1^ in their dry leaf tissue), and Ni (>1000 μg g^−1^ in their dry leaf tissue). For recent reviews of trace element hyperaccumulation, see Reeves (2003), Krämer (2010), van der Ent et al. (2013c, 2015e) and Pollard et al. (2014). Table 4 lists documented hyperaccumulator plants from the South and Southeast Asia region, listing the element hyperaccumulated, country of discovery, and relevant references. Figure 3 documents some of the nickel hyperaccumulator plants discovered from ultramafic soils in parts of South and Southeast Asia.

In one of the earliest geoecological studies of the region, Wither and Brooks (1977) and Brooks et al. (1977b) analysed herbarium samples of plants originating from Obi Island (North Moluccas). They identified *Myristica laurifolia* var. *bifurcata* (Myristicaceae), *Planchonella oxyhedra* (Sapotaceae), and *Trichospermum kjellbergii* (Malvaceae) as hyperaccumulators of Ni. The authors then analysed Ni concentrations in herbarium specimens of *T. kjellbergii* and *P. oxyhedra* from throughout their range in Southeast Asia and Oceania. The findings confirmed previously known ultramafic areas in Sulawesi and Indonesian New Guinea, as well as one in Ambon (South Moluccas) which was not documented on geological maps. Their suspicions about the substrate were confirmed by a 1994 geological study that mapped peridotite and serpentinite outcrops in both Ambon and Seram (Linthout and Helmers 1994). A more recent study in Soroako, Sulawesi, examined leaf tissue from 23 plant species from former Ni mining sites in search of hyperaccumulator plants (Netty et al. 2012). As a result, *Sarcotheca celebica* (Oxalidaceae) was confirmed as a Ni hyperaccumulator, with 1039 µg g^−1^ Ni in dry leaf tissue.

In a study describing the general influence of the ultramafic geochemistry on growth patterns of plants overlying two Malaysian massifs, the Bukit Rokan and Petasih along the Bentong-Raub suture zone on the Peninsula, Tashakor et al. (2013) document that the serpentinite of the area is strongly weathered and gives rise to characteristic red lateritic soils (Ferralsols). They point out that the greatest physiological stress experienced by plants growing on ultramafic soils is due to the low Ca: Mg ratio and the generally low available nutrients, and not due to potentially phytotoxic elements present in the soil, which are, for the most part, not in a plant-available form.

In a study of the Bela Ophiolite in the Wadh area of Balochistan, Pakistan, Naseem et al. (2009) discovered *Pteropyrum olivieri* (Polygonaceae) in a localized population over ultramafic soils. Although the plant did not hyperaccumulate, it had moderate concentrations of Ni, Co, and Cr in its tissues, typical of most plants growing on ultramafic soils.

The ultramafics of Malaysia and Indonesia have received considerable attention with regard to taxa with high metal-accumulating behavior. A chemical analysis of leaf litter from trees growing on ultramafics in Sabah, Malaysia (Proctor et al. 1989) confirmed that trees grow at low foliar nutrient concentrations and can concentrate Ca in their leaf tissue. Leaf litter showed an average Ca:Mg ratio as well as a high level of Ni, suggesting that senescence may act as a way of excreting excess Ni. From analysis of leaf litter, they found that *Shorea tenuiramulosa* (Dipterocarpaceae) and an unidentified species of *Syzygium* (Myrtaceae) accumulated Ni and Mn, respectively, with 1000 µg g^−1^ Ni and 13,700 µg g^−1^ Mn dry leaf weight. Proctor et al. (1994) also reported a yet to be named Ni-hyperaccumulating species of *Rinorea* from Mt Piapi on Karakelong Island, northeast of Sulawesi in Indonesia with up to 1830 µg g^−1^ foliar Ni.

In an analysis of 51 herbarium specimens from both Malaysia and Indonesia, including from Mount Kinabalu (Sabah), Soroako and Malili (Sulawesi) and Yapen Island, Reeves (2003) found high Ni values in *Phyllanthus insulae-japen* (Phyllanthaceae), which had been collected once in 1961, and in *R. bengalensis*, *Brackenridgea palustris* subsp. *kjellbergii* (Ochnaceae), *Glochidion* spp. (Phyllanthaceae), and two species of *Psychotria* (Rubiaceae) which could not be identified to species level. One ultramafic subspecies of *D. gelonioides* was identified as a Ni hyperaccumulator (subsp. *tuberculatum*), whereas another subspecies was confirmed as a Zn hyperaccumulator on non-ultramafic soils (subsp. *pilosum*) (Baker et al. 1992).

In recent studies of Mt. Kinabalu, van der Ent et al. (2013b, 2015a, f) discovered nine species of Ni hyperaccumulators from the flora of Kinabalu Park in Sabah, Malaysia. Previously known hyperaccumulators from the region included *R. bengalensis* (Brooks and Wither 1977a, b), *Rinorea javanica* (Brooks et al. 1977a), *P. balgooyi* (Phyllanthaceae; Hoffmann et al. 2003), *D. gelonioides* (Baker et al. 1992), *Psychotria* cf. *gracilis* (Rubiaceae; Reeves 2003), and *Shorea tenuiramulosa* (Proctor et al. 1989). Van der Ent et al. (2013b, 2015f) added several more Ni hyperaccumulators, including *Actephila alanbakeri* (*Cleistanthus* sp. nov. in the original report) (Phyllanthaceae; 11,520 µg g^−1^), *Flacourtia kinabaluensis* (Salicaceae; 7280 µg g^−1^), *Glochidion mindorense* (Phyllanthaceae; 2280 µg g^−1^), *Kibara coriacea* (Monimiaceae; 5840 µg g^−1^), *Mischocarpus sundaicus* (Sapindaceae) (4425 µg g^−1^), *Phyllanthus* cf. *securinegioides* (Phyllanthaceae; 23,300 µg g^−1^), *Psychotria sarmentosa* (Rubiaceae; 24,200 µg g^−1^), *Walsura pinnata* (Meliaceae; 4580 µg g^−1^), and *Xylosma luzoniensis* (Salicaceae; 5360 µg g^−1^) to the list, thereby documenting the highest number of Ni hyperaccumulators (15) known from any region within South and Southeast Asia.

In an effort to understand the factors contributing to Ni hyperaccumulation in Sabah, Malaysia, van der Ent et al. (2016b) examined the soil chemistry associated with 18 Ni hyperaccumulator plant species, comparing the chemistry of ultramafic soils where Ni hyperaccumulators were absent. The results showed that Ni hyperaccumulators are restricted to circum-neutral soils with relatively high phytoavailable Ca, Mg, and Ni. They hypothesized that either hyperaccumulators excrete large amounts of root exudates, thereby increasing Ni phytoavailability through intense rhizosphere mineral weathering, or that they have extremely high Ni uptake efficiency, thereby severely depleting Ni and stimulating re-supply of Ni via diffusion from labile Ni pools. Their results, however, tend to favor the latter hypothesis.

Nuclear microprobe imaging (micro-PIXE) shows that in *P. balgooyi* collected from ultramafic soils in Sabah, Malaysia, Ni concentrations were very high in the phloem of the stems and petioles, while in the leaves Ni was enriched in the major vascular bundles (Mesjasz-Przybylowicz et al. 2015). The preferential accumulation of Ni in the vascular tracts suggests that Ni is present in a metabolically active form. This research is important as the elemental distribution of *P. balgooyi* differs from that of many other Ni hyperaccumulators from temperate and Mediterranean regions where Ni is preferentially accumulated in leaf epidermal cells (Bhatia et al. 2004; Broadhurst et al. 2004; Tylko et al. 2007; Baklanov 2011).

In the Philippines, much of the ultramafic vegetation remains underexplored (Fernando et al. 2008; but see Baker et al. 1992; Fernando et al. 2013; Proctor et al. 1998, 2000a, b). Studies to date have revealed new Ni hyperaccumulators (e.g. Fernando and Rodda 2013; Hoffmann et al. 2003), including *Breynia cernua* (Phyllanthaceae; Gotera et al. 2014) and *P. balgooyi*, *P. erythrotrichus*, and *P. securinegioides* (Phyllanthaceae; Hoffmann et al. 2003; Quimado et al. 2015). A recent study described *Rinorea niccolifera* (Violaceae) as a novel taxon and Ni hyperaccumulator from Luzon Island, Philippines (Fernando et al. 2014).

Although in Sri Lanka’s ultramafic outcrops are not associated with many Ni hyperaccumulator species, unlike those in Sabah, Malaysia (van der Ent et al. 2015a), several plant species currently found at Ussangoda hyperaccumulate Ni (see citations in Chathuranga et al. 2015; Samithri 2015). Notable in this regard are *Evolvulus alsinoides* (Convolvulaceae), *Hybanthus enneaspermus* (Violaceae), *Flacourtia indica* (Flacourtiaceae), *Olax imbricata* (Olacaceae), *Toddalia asiatica* (Rutaceae), *Euphorbia heterophylla* (Euphorbiaceae), *Vernonia cinerea* (Asteraceae) and *Crotalaria* sp. (Fabaceae). Senevirathne et al. (2000) also document *Striga euphrasioides* (Orobanchaceae), *Cassia mimosoides* (Fabaceae), and *Blumea obliqua* (Asteraceae) from Ussangoda as hyperaccumulating Ni, although subsequent studies have failed to confirm this earlier report. Five Cu hyperaccumulators [*Geniosporum tenuiflorum* (Lamiaceae; now *Ocimum tenuiflorum*), *Clerodendrum infortunatum* (Lamiaceae), *Croton bonplandianus* (Euphorbiaceae), *Waltheria indica* (Malvaceae), and *Tephrosia villosa* (Fabaceae)] are also found on ultramafic outcrops in Sri Lanka (Rajakaruna and Bohm 2002). Based on revised criteria for Cu hyperaccumulation (van der Ent et al. 2013c), *Calotropis gigantea*, *Carissa spinarum*, *Cassia auriculata*, *Abutilon indicum*, and *Phyllanthus* sp. undet., analysed by Rajakaruna and Bohm (2002), now also qualify as hyperaccumulators of Cu (Table 4). Although Cu hyperaccumulation is not a common phenomenon among ultramafic plants, a recent study has also documented unusual Cu uptake in a number of ultramafic plants in Malaysia and Brazil (van der Ent and Reeves 2015).

**Table 4:** Unusual foliar elemental accumulation (Ni, Co, Cu, Mn or Zn—maximum recorded values in μg g^−1^) in plants from South and Southeast Asia

Family	Species	Life-form	Locality	Ni	Cu	Co	Mn	Zn	Reference
Acanthaceae	*Daedalacanthus suffruticosus*	Shrub	India	1235–1862	–	–	–	–	Datta et al. (2015)
Acanthaceae	*Ptyssiglottis* cf*. fusca*	Herb	Sabah, Malaysia	1160	–	–	–		Van der Ent et al. (2015f)
Amaranthaceae	*Aerva scandens*	Herb	Sulawesi, Indonesia	–	395	–	–	–	Brooks et al. (1978)
Amaranthaceae	*Cyathula prostrata*	Herb	Sulawesi, Indonesia	–	553	–	–	–	Brooks et al. (1978)
Apocynaceae	*Calotropis gigantea*	Climber	Sri Lanka	–	583	–	–	–	Rajakaruna and Bohm (2002)
Apocynaceae	*Carissa spinarum*	Climber	Sri Lanka	–	702	–	–	–	Rajakaruna and Bohm (2002)
Asteraceae	*Vernonia actaea*	Herb	Sulawesi, Indonesia	–	300	–	–	–	Brooks et al. (1978)
Asteraceae	*Vernonia cinerea*	Herb	Sri Lanka	1026	–	–	–	–	Samithri (2015)
Chrysobalanaceae	*Licania splendens*	Shrub	Zambales, Philippines	2728	–	–	–	–	Fernando et al. (2013)
Convolvulaceae	*Evolvulus alsinoides*	Herb	Sri Lanka	1478	–		–	–	Rajakaruna and Bohm (2002)
Dichapetalaceae	*Dichapetalum gelonioides* subsp*. pilosum*	Climber/shrub	Sabah, Malaysia	–	–	–	–	7000	Baker et al. (1992)
Dichapetalaceae	*Dichapetalum gelonioides* subsp*. sumatranum*	Shrub	SE Asia	–	–	–	–	30,000	Baker et al. (1992)
Dichapetalaceae	*Dichapetalum geloniodes* subsp*. tuberculatum*	Shrub	Malaysia and Philippines	26,600	–	–	–	–	Baker et al. (1992)
Dichapetalaceae	*Dichapetalum gelonioides* subsp. *andamanicum*	Shrub	Andaman Islands, India	3160; 9740–36,100	–	–	–	–	Brooks (1987), Datta et al. (2015)
Dipterocarpaceae	*Shorea tenuiramulosa*	Tree	Sabah, Malaysia	1790	–	–	–	–	Proctor et al. (1988a, b), Van der Ent et al. (2015a, b, c, d, e, f, g)
Euphorbiaceae	*Croton bonplandianus*	Tree	Sri Lanka	–	2163	–	–	–	Rajakaruna and Bohm (2002)
Euphorbiaceae	*Euphorbia thymifolia*	Shrub	Sri Lanka	1074	–	–	–	–	Samithri (2015)
Fabaceae	*Cassia auriculata*	Shrub	Sri Lanka	–	885	–	–	–	Rajakaruna and Bohm (2002)
Fabaceae	*Dalbergia beccarii*	Shrub	Sabah, Malaysia	2623	–	–	–	–	Van der Ent and Reeves (2015)
Fabaceae	*Tephrosia villosa*	Herb	Sri Lanka	–	1858	–	–	–	Rajakaruna and Bohm (2002)
Lamiaceae	*Clerodendrum infortunatum*	Herb	Sri Lanka	–	2278	–	–	–	Rajakaruna and Bohm (2002)
Lamiaceae	*Coleus scutellarioides*	Herb	Sri Lanka	–	500	–	–	–	Brooks et al. (1978)
Lamiaceae	*Ocimum tenuiflorum*	Herb	Sri Lanka	–	2266	–	–	–	Rajakaruna and Bohm (2002)
Loganiaceae	*Strychnos andamanensis*	Climber	India	2606–6893	–	–	–	–	Datta et al. (2015)
Loganiaceae	*Strychnos minor*	Climber	India	3220–10,214	–	–	–	–	Datta et al. (2015)
Loganiaceae	*Strychnos wallichiana*	Climber	India	2924–15,630	–	–	–	–	Datta et al. (2015)
Malvaceae	*Abutilon indicum*	Shrub	Sri Lanka	–	915	–	–	–	Rajakaruna and Bohm (2002)
Malvaceae	*Waltheria indica*	Shrub	Sri Lanka	–	1504	–	–	–	Rajakaruna and Bohm (2002)
Meliaceae	*Walsura monophylla*	Tree	Malaysia and Philippines	7090	–	–	–	–	Baker et al. (1992)
Meliaceae	*Walsura pinnata*	Tree	SE Asia	4580	–	–	–	–	Van der Ent et al. (2015f)
Monimiaceae	*Kibara coriacea*	Tree	SE Asia	5840	–	–	–	–	Van der Ent et al. (2015f)
Moraceae	*Ficus brevicuspis*	Tree	India	28,322–30,564	–	–	–	–	Datta et al. (2015)
Myristicaceae	*Knema matanensis*	Tree	Indonesia	5000	–	–	–	–	Van der Ent et al. (2013a)
Myristicaceae	*Myristica laurifolia* var*. bifurcata*	Tree	Indonesia	1100	–		–	–	Wither and Brooks (1977)
Myrtaceae	*Decaspermum blancoi*	Shrub	Zambales, Philippines	1996	–		–	–	Fernando et al. (2013)
Ochnaceae	*Brackenridgea palustris* subsp. *foxworthyi*	Shrub	Philippines	7600	–	–	–	–	Baker et al. (1992)
Ochnaceae	*Brackenridgea palustris* subsp*. kjellbergii*	Tree	Sulawesi, Indonesia	1440	–	–	–	–	Reeves (2003)
Ochnaceae	*Ochna integerrima*	Tree	India	2465–5210	–	–	–	–	Datta et al. (2015)
Olacaceae	*Olax imbricata*	Tree	Sri Lanka	1082	–	–	–	–	Samithri (2015)
Oxalidaceae	*Sarcotheca celebica*	Tree	Indonesia	1000	–	–	–	–	Van der Ent et al. (2013a, b, c)
Papilionaceae	*Cassia sophera*	Shrub	Sulawesi, Indonesia	–	333	–	–	–	Brooks et al. (1978)
Phyllanthaceae	*Actephila alanbakeri*	Shrub	Sabah, Malaysia	11,520	–	–	–	–	Van der Ent et al. (2016c)
Phyllanthaceae	*Aporosa chalarocarpa*	Tree	SE Asia	1560	–	–	–	–	Van der Ent et al. (2015f)
Phyllanthaceae	*Baccaurea lanceolata*	Tree	SE Asia	1450	–	–	–	–	Van der Ent et al. (2015f)
Phyllanthaceae	*Breynia cernua*	Shrub	Zambales, Philippines	3573	–	–	–	–	Gotera et al. (2014)
Phyllanthaceae	*Cleistanthus* sp. 1	Tree	Sabah, Malaysia	2110	–	–	–	–	Van der Ent et al. (2015f)
Phyllanthaceae	*Glochidion* aff*. acustylum*	Tree	Sulawesi, Indonesia	6060	–	–	–	–	Reeves (2003)
Phyllanthaceae	*Glochidion brunneum*	Tree	SE Asia	6200	–	–	–	–	Van der Ent et al. (2015f)
Phyllanthaceae	*Glochidion* cf*. lanceisepalum*	Tree	Sabah, Malaysia	3270	–	–	–	–	Van der Ent et al. (2015f)
Phyllanthaceae	*Glochidion* cf. *mindorense*	Tree	SE Asia	2280	–	–	–	–	Van der Ent et al. (2015f)
Phyllanthaceae	*Glochidion* cf. *rubrum*	Tree	SE Asia	7000	–	–	–	–	Van der Ent et al. (2015f)
Phyllanthaceae	*Glochidion* cf. *sericeum*	Tree	Sabah, Malaysia	2190	–	1310	–	–	Van der Ent et al. (2015f); Van der Ent (unpublished)
Phyllanthaceae	*Glochidion* sp. ‘bambangan’	Tree	Sabah, Malaysia	16,700	–	–	–	–	Van der Ent et al. (2015f)
Phyllanthaceae	*Glochidion* sp. ‘nalumad’	Tree	Sabah, Malaysia	9000	–	–	–	–	Van der Ent et al. (2015f)
Phyllanthaceae	*Phyllanthus balgooyi*	Tree	Malaysia and Philippines	8610	–	–	–	–	Hoffmann et al. (2003), Mesjasz-Przybylowicz et al. (2015)
Phyllanthaceae	*Phyllanthus erythrotrichus*	Shrub	Zambales, Philippines	17,520	–	–	–	–	Quimado et al. (2015)
Phyllanthaceae	*Phyllanthu*s *securinegioides*	Shrub	Sabah, Malaysia	23,300	–	–	–	–	Baker et al. (1992), Van der Ent et al. (2015f)
Phyllanthaceae	*Phyllanthus* sp. undet.	Shrub	Sri Lanka	–	821	–	–	–	Rajakaruna and Bohm (2002)
Piperaceae	*Peperomia pellucida*	Shrub	Sulawesi, Indonesia	–	300	–	–	–	Brooks et al. (1978)
Rubiaceae	*Psychotria* cf*. gracilis*	–	Sabah, Malaysia	10,590	–	–	–	–	Reeves (2003)
Rubiaceae	*Psychotria sarmentosa*	Climber	Sabah, Malaysia	24,200	–	–	–	–	Van der Ent et al. (2015f)
Rubiaceae	*Psychotria* sp. undet.	–	Sulawesi, Indonesia	1820	–	–	–	–	Reeves (2003)
Rubiaceae	*Urophyllum* cf. *macrophyllum*	Herb	Sabah, Malaysia	–	–	–	10,464	–	Van der Ent and Reeves (2015)
Salicaceae	*Flacourtia indica*	Tree	Sri Lanka	1165	–	–	–	–	Samithri (2015)
Salicaceae	*Flacourtia kinabaluensis*	Tree	Sabah, Malaysia	7280	–	–	–	–	Van der Ent et al. (2015f)
Salicaceae	*Xylosma luzonensis*	Tree	SE Asia	5360	–	–	–	–	Van der Ent et al. (2015f)
Sapindaceae	*Mischocarpus sundaicus*	Tree	SE Asia	4425	–		–	–	Van der Ent et al. (2015f)
Sapotaceae	*Planchonella obovata*	Tree	Zambales, Philippines	1005	–	–	–	–	Fernando et al. (2013)
Sapotaceae	*Planchonella oxyedra*	Tree	Obi Island, Indonesia	19,600	–	–	–	–	Wither and Brooks (1977)
Tiliaceae	*Trichospermum kjellbergii*	Tree	Indonesia	3770	–	–	–	–	Wither and Brooks (1977)
Urticaceae	*Laportea ruderalis*	Herb	Sulawesi, Indonesia	–	600	–	–	–	Brooks et al. (1978)
Verbenaceae	*Callicarpa* sp. undet.	Shrub	Zambales, Philippines	1052	–	–	–	–	Fernando et al. (2013)
Violaceae	*Hybanthus enneaspermus*	Shrub	Sri Lanka	1862	–	–	–	–	Rajakaruna and Bohm (2002)
Violaceae	*Rinorea bengalensis*	Tree	S & SE Asia and Australia	2723–18,840	–	–	–	–	Brooks and Wither (1977); Datta et al. (2015)
Violaceae	*Rinorea javanica*	Tree	SE Asia	9680	–	–	–	–	Brooks and Wither (1977)
Violaceae	*Rinorea niccolifera*	Shrub	Luzon Island, Philippines	18,388	–	–	–	–	Fernando et al. (2014)
Violaceae	*Rinorea* sp. nov.	Shrub	Talaud Island, Indonesia	1830	–	–	–	–	Proctor et al. (1994)

## Evolutionary aspects

Ultramafic outcrops often harbor populations which are morphologically and physiologically distinct from those found on non-ultramafic soils. Such intraspecific variation, especially with respect to functionally important traits, is common in many ultramafic taxa worldwide (O’Dell and Rajakaruna 2011). Such variation can result from both local adaptation (i.e., ecotypic differentiation; Sambatti and Rice 2006; Turner et al. 2010) or phenotypic plasticity (Murren et al. 2006; Wu et al. 2010), and must be examined on a case-by-case basis. Suitable methods of examination include reciprocal or unilateral transplant experiments and common garden studies (Wright and Stanton 2011), as well as functional genomic and proteomic approaches (Selby et al. 2014; von Wettberg et al. 2014; von Wettberg and Wright 2011). Detecting intraspecific variation is the first step toward any investigation on the causes and consequences of adaptive evolution. Populations exhibiting intraspecific variation on ultramafic and non-ultramafic soils have led to numerous studies of speciation (Anacker 2014; Kay et al. 2011) and phylogenetic investigations (Anacker 2011; Anacker et al. 2011; Anacker and Harrison 2012), advancing our understanding of evolutionary and ecological theory (Harrison and Rajakaruna 2011). Molecular phylogenetic methods provide a unique protocol for testing and establishing species relationships, helping to shed light on how ultramafic endemics evolve (Baldwin 2005). The analysis of phylogenies for 23 genera from California shows that ultramafic endemics exhibit few transitions out of the endemic state (Anacker et al. 2011), suggesting that adaptation to ultramafics and subsequent diversification can lead to an evolutionary “dead end”. But ultramafic lineages may not always represent evolutionary “dead ends” and may have the potential to further diversify via independent polyploidization and hybridization, even providing a pathway to radiate off ultramafic soils (Kolář et al. 2012).

Compared to these studies from other regions of the world, there is little information on evolutionary aspects of plants associated with ultramafic soils in South and Southeast Asia. A recent study from Sri Lanka shows that the ultramafic and non-ultramafic populations of *Fimbristylis ovata* (Cyperaceae) may be locally adapted to their respective soils (Chathuranga et al. 2015). The ultramafic population translocated significantly more Ni from its roots to shoots (translocation factor 0.43) than the non-ultramafic population (translocation factor 0.29). However, additional studies are required to determine whether the populations of *F. ovata*, or other species, including those hyperaccumulating metals such as Ni and Cu, deserve ecotypic recognition. Several ultramafic-associated taxa in Sri Lanka might benefit from further observations and additional greenhouse studies to determine whether the ultramafic-associated populations are genetically distinct and are worthy of ecotypic recognition (Rajakaruna and Bohm 2002). These taxa include several Ni-accumulating and -hyperaccumulating species, particularly *Hybanthus enneaspermus* (Violaceae), *Evolvulus alsinoides* (Convolvulaceae), *Crotalaria* sp. (Fabaceae), *Desmodium triflorum* (Fabaceae) and *Fimbristylis* sp. (Cyperaceae), all of which show detectable phenotypic differences between ultramafic and non-ultramafic populations. Studies exploring causes and consequences of phenotypic differences between populations found on and off ultramafic soils can add much to our understanding of the origins of ultramafic specialists in the South and Southeast Asia region.

## Phytotechnologies

The use of trace element hyperaccumulators to clean up polluted sites, i.e. phytoremediation, is gaining recognition as a viable green technology (Neilson and Rajakaruna 2014). Phytoremediation is based on the premise that plants which remove selected pollutants from the soil and translocate them to their above-ground biomass can then be harvested and disposed of through incineration or elemental recovery, a process known as phytomining (Chaney et al. 2014; van der Ent et al. 2015g). Ultramafic plants in the genera *Alyssum* (Brassicaceae), *Streptanthus* (Brassicaceae), *Noccaea* (Brassicaceae), and *Berkheya* (Asteraceae) have been used in phytoremediation and phytomining of Ni-enriched ultramafic sites in temperate and Mediterranean regions (Ho et al. 2013; Morel et al. 2006; Gall and Rajakaruna 2013; Sheoran et al. 2009; van der Ent et al. 2015g). Given the large number of hyperaccumulator species currently known from tropical Asia (Gall and Rajakaruna 2013; Reeves 2003), there should be considerable interest in using these unique plants in the remediation of regional sites contaminated with metal and metalloid pollutants.

### Phytoremediation and phytomining

Bandara et al. (2017) investigated the effect of biochar and fungal-bacterial co-inoculation on soil enzymatic activity and immobilization of heavy metals in soil collected from an ultramafic outcrop in Sri Lanka. The addition of biochar to ultramafic soil immobilized heavy metals and decreased soil enzymatic activities while the addition of microbial inoculants improved plant growth by mitigating heavy metal toxicity and enhancing soil enzymatic activities. Additional studies from Sri Lanka confirm the importance of (i) bacterial-fungal inoculation as a soil-quality enhancer and a plant-growth promoter in the presence of heavy metals found in ultramafic soils (Seneviratne et al. 2016a, b), and, (ii) biochar as a soil amendment to immobilize Cr, Ni, and Mn in ultramafic soil, thereby reducing metal-induced plant toxicities (Herath et al. 2014).

The potential for microbial remediation (reduction) of Cr(VI) by indigenous microbial populations from the ultramafic soils of Sukinda mines in Jaipur, Orissa, India, was investigated by Mishra et al. (2009). The best reducer of Cr (V1) was *Staphylococcus aureus*, a gram-positive bacterium whose thick layer of peptidoglycan acts as a strong absorbent. The taxon tolerated a Cr concentration of 250 mg L^−1^ and was resistant to Ni up to 1000 mg L^−1^. The bacterium was recommended for the bioremediation of both Cr and Ni, showing complete Cr(VI) to Cr(III) degradation in 22 h, and Ni^2+^ degradation to 90% in 22 h. Similarly, Bohidar et al. (2009) explored the possibility of Ni recovery from chromite tailings at the Sukinda mines by using three fungal strains.

In another study, Mohanty et al. (2011) utilized phytoremediation in South Kaliapani, a chromite mining ultramafic area in Orissa, India. Chromium was extracted by growing *Oryza sativa* cv. Khandagiri (rice; Poaceae) in contaminated soil and irrigating with mine wastewater. Chromium levels were reduced (70–90%) after 100 days, with accumulation levels ranging from 125 to 498 µg g^−1^ in leaves, 25 to 400 µg g^−1^ in stems, and 5 to 23 µg g^−1^ in the grain. Absorption into roots was higher by two orders of magnitude than into any aerial part of the plant. Mohanty et al. (2012) also investigated the phytoremediation potential of *O. sativa*, *Brachiaria mutica* (Poaceae), and *Eichhornia crassipes* (Pontederiaceae) to reduce levels of Cr(VI) in mine waste-water. *Eichhornia crassipes* was most successful with 25–54% reduction while *B. mutica* contributed to an 18–33% reduction.

Kfayatullah et al. (2001), in a study of plants and soils of the Malakand chromite-rich ultramafic area and Mardan non-ultramafic areas of the North-West Frontier Province, Pakistan, focused on enzyme-bound metal accumulation in plant tissue. *Verbascum thapsus* (Scrophulariaceae), an edible plant, accumulated greater than 100 µg g^−1^ of several metals, including Ni and Cr, but was not recommended for phytoremediation efforts.

Indonesia (Sulawesi and Halmahera Islands) has some of the largest surface exposures of ultramafic bedrock in the world. Lateritic Ni-mining operations have continued in the region since the early twentieth century, setting the stage for exploring the use of native plants for phytoremediation and phytomining. Twelve native species known to hyperaccumulate Ni are recommended by van der Ent et al. (2013a) for use in phytotechnologies in Indonesia.

## Threats and conservation

Ultramafic areas are a high priority for biodiversity conservation because of the relatively large numbers of endemic species, ecotypes, and rare species that they harbour (Boyd et al. 2009). The conservation and restoration of these naturally fragmented, edaphically unique, and biodiverse habitats require special attention (Baker et al. 2010; O’Dell 2014; Thorne et al. 2011; Whiting et al. 2004). It is unclear how stressors, such as atmospheric N deposition (Vallano et al. 2012), suppression of fire (Arabas 2000; Safford and Harrison 2004) and climate change (Damschen et al. 2012; Anacker and Harrison 2012) documented for temperate and Mediterranean ultramafics, impact tropical Asia’s ultramafic ecosystems.

The combined forces of forest clearing, agricultural development and mining contribute to unprecedented habitat loss in South and Southeast Asia (Duckworth et al. 2012; Hughes 2017; Sodhi et al. 2004). In fact, Southeast Asia has a higher annual rate of deforestation than Meso-America, South America, or sub-Saharan Africa, and that rate has continued to increase between 1990 and 2005 (Giam et al. 2010; Sodhi et al. 2010). This is especially of concern as Southeast Asia has a higher proportion of its vascular plant, reptile, bird, and mammal species categorised as globally threatened on the Red List compared to Meso- and South America and sub-Saharan Africa (Sodhi et al. 2010). With such limited study of ultramafics in South and Southeast Asia, it is unclear how increasing habitat loss is impacting biodiverse ultramafic outcrops in the region.

**Fig. 4:**
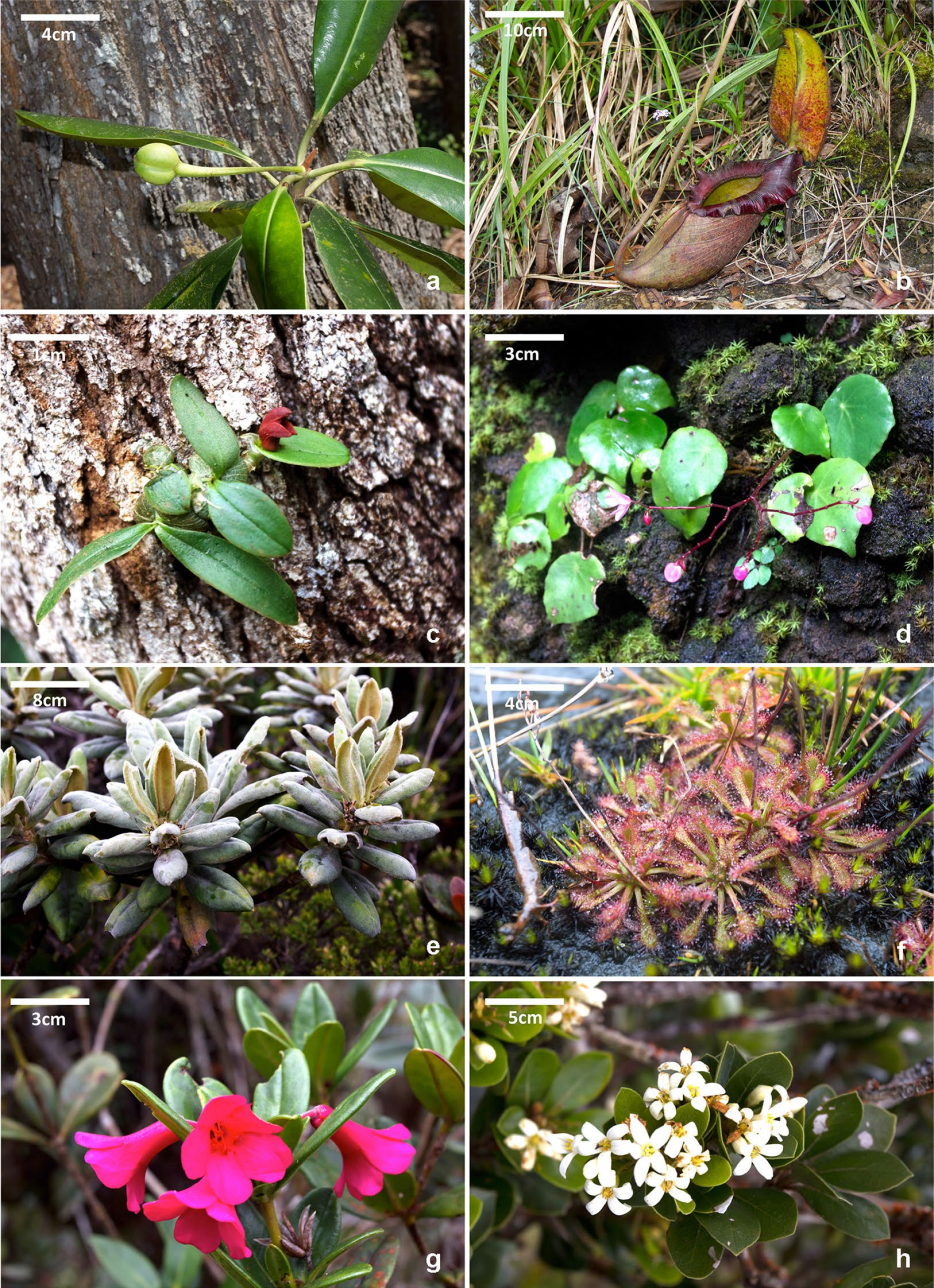
Ultramafic edaphic endemics from South and Southeast Asia: **a** The monotypic tree *Borneodendron aenigmaticum* (Euphorbiaceae) is endemic to Sabah (Malaysia) on ultramafic soils in the lowlands. **b** The world’s largest carnivorous pitcher plant, *Nepenthes rajah* (Nepenthaceae) is endemic to Kinabalu Park in Sabah where it occurs in the montane zone. **c** The epiphytic or lithophytic orchid *Porpax borneensis* (Orchidaceae) is restricted to ultramafic outcrops in Sabah, Malaysia. **d** The recently described *Begonia moneta* (Begoniaceae) occurs lithophytically in lowland ultramafic forest in Sabah, Malaysia. **e** *Scaevola verticillata* (Goodeniaceae) is endemic to the summit of the ultramafic Mount Tambukon in Sabah, Malaysia. **f** The carnivorous *Drosera ultramafica* (Droseraceae) is endemic to a limited number of mountainous ultramafic outcrops in Malaysia and the Philippines. **g** *Rhododendron baconii* (Ericaceae) is another hyper-endemic restricted to Kinabalu Park, Sabah, Malaysia. **h** The specific epithet of *Pittosporum peridoticola* (Pittosporaceae) indicates its habitat is on ultramafic soils in Sabah, Malaysia (all images are by A. van der Ent)

Malaysia has one of the most species-rich ultramafic floras in the world. The over 3500 km^2^ of ultramafic outcrops in Sabah (4.6% of the total landmass of the state) on the island of Borneo harbor a total of 4252 plant species (van der Ent et al. 2015a). Over 2542 plant species have been documented on ultramafic outcrops in Kinabalu Park alone, of which a large percentage is endemic to either Kinabalu Park or to Borneo (van der Ent et al. 2015a; Fig. 4). Despite the existence of this species-rich flora, the plant diversity and ecology of many ultramafic outcrops in Sabah remain largely unknown because of a lack of focused research. Furthermore, plant diversity in many areas of Sabah is severely threatened by land-use conversion and, because often plant species occur only at a single or a few ultramafic sites, and hence impacts on the ecosystems that support them could eventually result in their extinction. While it is necessary to identify stressors impacting ultramafic habitats of South and Southeast Asia for their proper management, it is even more critical that basic geoecological surveys of ultramafic outcrops, including the extensive exposures in Sulawesi and Halmahera, are prioritised for cataloguing plant diversity and other biota. This is especially critical as many of these outcrops likely harbor rare and endemic species in need of urgent conservation attention.

Although Sri Lanka’s ultramafic flora appears to be impoverished with respect to endemic species or hyperaccumulator taxa, the ultramafic sites harbor several taxa worthy of conservation. For example, Ussangoda, the site that has received the most research attention, is home to: four near-threatened species, *Striga angustifolia* (Orobanchaceae), *Maerua arenaria* (Capparaceae), *Salvadora percia* (Salvadoraceae), and *Olax imbricata* (Olacaceae); two vulnerable species, *Cyanotis adscendens* (Commelinaceae), *Pachygone ovata* (Menispermaceae); and one data deficient species, *Alysicarpus monilifer* (Fabaceae; MOE 2012). Therefore, it is critical that Sri Lanka’s ultramafic outcrops receive regional and national recognition and are declared as ecologically sensitive sites (i.e. geoecological preserves) to be set aside for future investigations. In 2010, Ussangoda was declared as a National Park with approximately 350 hectares, including areas overlaying ultramafic rock, set aside for conservation purposes (Department of Wildlife Conservation 2015). Without such conservation, proper management, and research, these unique habitats and their physiologically distinct biota are extremely vulnerable. *Rinorea bengalensis* (Violaceae) offers an example of why such efforts are urgently needed. Brooks et al. (1977a, b) conducted a survey of herbarium specimens from the entire range of this species, encompassing Sri Lanka, the Malay Archipelago, New Guinea, the Solomon Islands and Queensland, Australia, and found that Ni hyperaccumulation is a constitutive trait in this species when growing on ultramafic soil. The herbarium specimen analysed from Sri Lanka contained 10,000 µg g^−1^ and the locality indicated on the map presented by Brooks et al. (1977a) suggests a collection in the central part of the island (see Fig. 1 in Rajakaruna and Baker 2004). However, it was not encountered in field exploration by Rajakaruna and Bohm (2002) and was presumed extinct in Sri Lanka (Ministry of Environment and Renewable Energy 2012). Interestingly, the taxon was recently recollected in southwestern Sri Lanka (Siril Wijesundara, National Institute of Fundamental Studies, Sri Lanka, pers. comm.), however, soil and plant tissue elemental concentrations have yet to be determined.

## Conclusions

### Information gaps and future directions

Ultramafic outcrops are natural laboratories for experimental and applied research in a wide range of disciplines. They provide numerous opportunities for collaborations among geologists, pedologists, botanists, zoologists, microbiologists, and land managers focusing on conservation and restoration research. However, research on the ultramafic outcrops in South and Southeast Asia has been limited, with most effort to date focused on Malaysia, the Philippines, the Andaman Islands (India), and Sri Lanka (Table 1). We were unable to find any published literature on ultramafic geoecology of other South (Afghanistan, Bhutan, Nepal) and Southeast Asian (Myanmar, Laos, Thailand, Vietnam) countries despite the known occurrences of ultramafic lithologies in these locales. The limited number of published studies we found for Myanmar, Thailand, and Vietnam (Table 1) focused on geological, mineralogical, or geochemical research.

Throughout South and Southeast Asia, detailed and systematic surveys will likely reveal numerous species new to science, including trace element hyperaccumulators. Recent research conducted in Sabah, Malaysia by van der Ent et al. (2014, 2015a, f) which led to the discovery of 24 new hyperaccumulator species, is a case in point. Detailed floristic surveys should be undertaken across the region and species showing unusual physiological behavior (such as trace element accumulation) or exhibiting distinct morphological traits relative to populations on non-ultramafic soils may be further studied under laboratory and greenhouse conditions. Additionally, species showing intraspecific variation between ultramafic and non-ultramafic populations may be evaluated via population genetic studies to determine whether ultramafic populations are genetically distinct from those found on non-ultramafic soils. For those species showing intraspecific variation with respect to morphological or physiological features, including flowering times between ultramafic and non-ultramafic populations, common garden and reciprocal transplant experiments can be undertaken to examine whether populations are locally adapted to their substrate. Such types of experimental studies are currently lacking entirely from the region.

In addition to detailed studies of vascular plants, it is important to pay close attention to non-vascular plants such as bryophytes, cryptogamic species such as lichens, soil algae and cyanoprokaryotes, and belowground microbes and soil invertebrates. Such investigations will likely reveal species that are endemic to the substrate or show a high affinity to ultramafic soils, as shown for such research conducted in South Africa (Venter et al. 2015) and California, USA (Rajakaruna et al. 2012).

Species documented as trace element hyperaccumulators may be investigated under controlled conditions for their suitability for phytoremediation or phytomining and tested under field conditions for their effectiveness in site reclamation and restoration. The resulting information can be added to the global database of metal hyperaccumulating species (Global Hyperaccumulator Database 2016: http://www.hyperaccumulators.org). Finally, it is critical that tropical Asia’s ultramafic outcrops receive regional, national, and global recognition and that key sites receive appropriate statutory protection so that future scientific research is possible.

One of the options for protection at a national level by the state is the inclusion of ultramafic sites in the Global Geopark Network (GGN). Conservation and protection of landscapes of geological significance at a national and international level is promoted by UNESCO under its Global Geoparks Scheme (UNESCO 2016). At a national level, relevant authorities should pursue this option as a long-term conservation strategy, which would provide a holistic approach to protection by incorporating a management strategy including education and sustainable development. The latter would mobilize the local population for economic benefits by participating in the conservation efforts through local and international ecotourism. This, however, also requires meeting the stringent guidelines laid out by UNESCO to be included in the GGN. Currently, ultramafic sites in South and Southeast Asia are not in the GGN but would meet the basic requirements laid out by UNESCO.
